# Hospitals Closed for Repairs

**Published:** 1892-09-03

**Authors:** 


### Passing Topics.

### HOSPITALS CLOSED FOR REPAIRS.

The closing entirely or partially of several of the metropolitan hospitals at one and the same time for the very necessary operations of cleansing and repair appears to have aroused a somewhat strong feeling of disapproval in certain quarters. As usual, there is something to be said upon both sides, but if the facts are carefully weighed, it will be found that the balance of argument is in favour of the institutions rather than of the complainants.

The circumstance that Charing Cross and University College Hospitals should be wholly closed, and that another hospital—the Middlesex—situate somewhere between the two should be working with less than half its beds available, is no doubt unfortunate, but we think the most to be said in that connection is, that the inconvenience to the public would have been diminished, if not altogether removed, had the hospitals only seen their way to communicate with each other before their several plans were finally decided upon. Now here is an illustration of something which ought not to be, and is so easily preventible by intercommunication, that the advocates of a central consultative body might well adduce it in support of their demands. With the premisses once admitted, as admitted they must be, it would be difficult for even the most litigious to deny the conclusion.

But in regard to the time of closing, it may be pointed out that the periodical cleansing of a hospital ward is indispensable to the wellbeing of the patients who are to occupy it. As a matter of fact, an arrangement whereby every ward and ward accessory existed in duplicate would be an exceedingly desirable one if it were possible. By that means the occupants could be turned over at regular intervals to new quarters, and the wards evacuated would lie fallow until the process needed to be repeated. At present wards are frequently cleansed in hot haste, and the patients are re-admitted without the loss of a moment, simply because the pressure is too great to allow of a reasonable rest. At Charing Cross, besides more ordinary work, we believe that important improvements have been carried out in the system of drainage, and surely the most exacting of critics will neither deny the necessity for closing, nor contend that a period of less than seven weeks is any too long for operations of that character.

As to the time chosen for the work, the hospitals might fairly ask what part of the year would be preferable. Quite irrespective of any superfluous tenderness for the medical schools, which, as it appears to us, have been somewhat unfairly involved in this question, every manager would agree that if there be a quiet season in the hospital year it may be looked for in August and September more commonly than at any other time.

The hospitals, even the best abused of them, may take heart of grace. It is some testimony to their value that they are so much in request. No feature of the situation is more instructive than that the temporary closing of the hospitals, even for indispensable purposes, is considered a calamity by people who perhaps are unable to show their appreciation of these institutions in any other way. But do these solicitous critics remember that a large number of beds are more or less permanently lost to the sick poor because there are no means to maintain them? These are the gentlemen who strain at a gnat and swallow a camel. The loss of the services of Charing Cross and University College Hospitals during a few weeks fills them with dismay. What are they prepared to do in order that Guy's and St. Thomas's may throw open the wards which have stood empty for years?

Agricola.

